# Monitoring the Effectiveness of Treatment in Women with Schizophrenia: New Specialized Cooperative Approaches

**DOI:** 10.3390/brainsci13091238

**Published:** 2023-08-25

**Authors:** Mentxu Natividad, Mary V. Seeman, Jennipher Paola Paolini, Ariadna Balagué, Eloïsa Román, Noelia Bagué, Eduard Izquierdo, Mireia Salvador, Anna Vallet, Anabel Pérez, José A. Monreal, Alexandre González-Rodríguez

**Affiliations:** 1 Department of Mental Health, Mutua Terrassa University Hospital, Fundació Docència i Recerca Mutua Terrassa, University of Barcelona, 08221 Terrassa, Spain; mnatividad@mutuaterrassa.cat (M.N.); jpaolini@mutuaterrassa.cat (J.P.P.); eloisaroman@mutuaterrassa.cat (E.R.); nbague@mutuaterrassa.cat (N.B.); eizquierdo@mutuaterrassa.cat (E.I.); msalvador@mutuaterrassa.cat (M.S.); avallet@mutuaterrassa.cat (A.V.); aperez@mutuaterrassa.cat (A.P.); 2 Department of Psychiatry, University of Toronto, Toronto, ON M5P 3L6, Canada; mary.seeman@utoronto.ca; 3 Institut de Neurociències, Universitat Autònoma de Barcelona (UAB), 08221 Terrassa, Spain; 4 Centro de Investigación Biomédica en Red de Salud Mental (CIBERSAM), 28029 Madrid, Spain

**Keywords:** schizophrenia, psychosis, women, specialized care, observatories

## Abstract

Women with schizophrenia have specific health needs that differ from those of men and that change through successive life stages. We aimed to review the biopsychosocial literature on schizophrenia that addresses clinically important questions related to the treatment of women, including somatic morbi-mortality, hyperprolactinemia, comorbid substance use disorders, social risk factors, and medication effectiveness/safety. Data search terms were as follows: (Morbidity AND mortality) OR hyperprolactinemia OR (“substance use disorders” OR addictions) OR (“social risk factors”) OR (“drug safety” OR prescription) AND women AND schizophrenia. A secondary aim was to describe a method of monitoring and interdisciplinary staff strategies. Schizophrenia patients show an increased risk of premature death from cardiovascular/respiratory disease and cancer compared to the general population. The literature suggests that close liaisons with primary care and the introduction of physical exercise groups reduce comorbidity. Various strategies for lowering prolactin levels diminish the negative long-term effects of hyperprolactinemia. Abstinence programs reduce the risk of victimization and trauma in women. Stigma associated with women who have serious psychiatric illness is often linked to reproductive functions. The safety and effectiveness of antipsychotic drug choice and dose differ between men and women and change over a woman’s life cycle. Monitoring needs to be multidisciplinary, knowledgeable, and regular.

## 1. Introduction

Male/female differences are well known to exist in schizophrenia [1]. For instance, negative and cognitive symptoms are more frequent in men, and positive psychotic and affective symptoms more prevalent in women of reproductive age [2]. Women, as a group, show an overall superior response to antipsychotic treatment compared to men, but they develop more adverse effects [3]. Gender/sex are important variables that influence the clinical course of schizophrenia and determine total clinical health needs [1]. Physical as well as emotional/mental health needs differ in men and women with schizophrenia and these needs change, in women, pre and post menopause [1]. One of the most significant sex differences is that men show psychotic symptoms earlier in life. On the other hand, there is a second peak of new onset schizophrenia, in women only, at the time of menopause, coincident with estrogen decline [1,2,3].

The findings cited above remain somewhat inconclusive in the literature, perhaps because clinical trials tend to recruit relatively few women [4]. Despite this selection bias, the vast majority of studies support the hypothesis that female sex hormones improve the response to specific antipsychotic drugs and that the addition of selective estrogen receptor modulators (SERMs) to standard treatment results in clinical benefits [5,6].

A major global health problem is women’s poor access to medical care in some parts of the world [7], which is especially problematic because women are prone to medical comorbidities not commonly seen in men [1]. One example of these differences is the high prevalence of autoimmune disease in women compared to men [8,9] and the association of some of these immunologic diseases with schizophrenia [8,10]. Moreover, antipsychotic medications increase the rate of other diseases that occur frequently in women, e.g., osteoporosis, breast cancer, and obesity [4,11].

When women with schizophrenia reach menopause, they experience heightened medical comorbidity that is partly attributable to age but also due to estrogen depletion, socioeconomic factors, and lifestyle factors [12]. More specifically, women at this time show increased rates of substance use disorders, sleep apnea, hypercholesterolemia, venous thrombosis, and diabetes, as well as obesity and osteoporosis [12].

Our team previously published a description of a unit for the treatment of women with schizophrenia [1], which was newly established to address women-specific care needs. In inaugurating the unit, in addition to standard surveillance of the quality of care and suicide prevention, we put in place five treatment effectiveness monitoring stations or “observatories” for the following areas: (1) for somatic morbidity and mortality, (2) for hyperprolactinemia, (3) for substance use disorders, (4) for the presence of social exclusion or discrimination, and (5) for optimal drug prescribing and safety. Monthly staff meetings are now dedicated to reviewing clinical observations in these five problem areas. During the meetings, the following procedures are followed: (A) all deaths are reviewed, and causes/errors analyzed; (B) prolactin level monitoring is reviewed, with input from primary care and endocrinology; (C) substance abuse dilemmas are discussed with addiction specialists; (D) social determinants of health are debated among staff nurses, social workers, occupational therapists, psychologists, sociologists, and housing and employment experts; and (E) treatment resistant cases are reviewed and decisions made about the initiation of clozapine and the remediation of extrapyramidal and cardiometabolic adverse drug effects and drug–drug interactions. 

### Aims

The aim of this review is to explore the literature on the five issues relevant to our newly initiated observatories. 

## 2. Methods

### 2.1. Search Strategy

A narrative review was conducted by searching PubMed from January 2013 to June 2023. The following search terms were used: (morbidity AND mortality) OR hyperprolactinemia OR (“substance use disorders” OR addictions) OR (“social risk factors”) OR (“drug safety” OR prescription) AND women AND schizophrenia. Additional relevant papers from the reference lists of included papers were collected. The search was conducted by M.N., MV.S., and A.G.R. 

#### 2.1.1. Inclusion and Exclusion Criteria

Papers were included if they met the following criteria: (1) the paper was written in English, Spanish, French, or German, (2) the study included a report on somatic morbi-mortality/hyperprolactinemia/substance use disorders/social exclusion or discrimination or drug safety/effectiveness, (3) study participants were women with schizophrenia or related psychosis, and (4) the paper was published in a peer-reviewed journal. Exclusion criteria were as follows: studies based on expert opinions or case reports and papers inadequately reporting health outcomes. 

#### 2.1.2. Data Collection and Extraction

Relevant titles and abstracts were manually screened by two authors (M.N. and A.G.R.) and the search was expanded by the remaining authors. We then screened the selected full-text documents in search of additional eligible studies. Findings were arranged under our pre-determined search categories and are presented in the text and in Table 1.

### 2.2. Monitoring Stations or Clinical “Observatories”

The Mutua Terrassa Functional Unit for Women with Schizophrenia is a pilot project of a community mental health service that was initiated in January 2023 [1]. Its main goal is improving physical and mental health and identifying social determinants of health in women suffering from schizophrenia and related disorders. The unit inaugurated five working “observatories” of health to improve clinical care. These were defined as cooperative strategies to prevent, detect, monitor, and treat specific adverse events (morbi-mortality, hyperprolactinemia, comorbid substance use disorders, social exclusion/discrimination, and drug resistance or negative side effects).

The observatories meet monthly to discuss all deaths, all cases of hyperprolactinemia, severe substance use disorders, social exclusion/discrimination, and treatment resistance/adverse drug events. The present review is intended to help with staff training. 

## 3. Results

**Figure 1:**
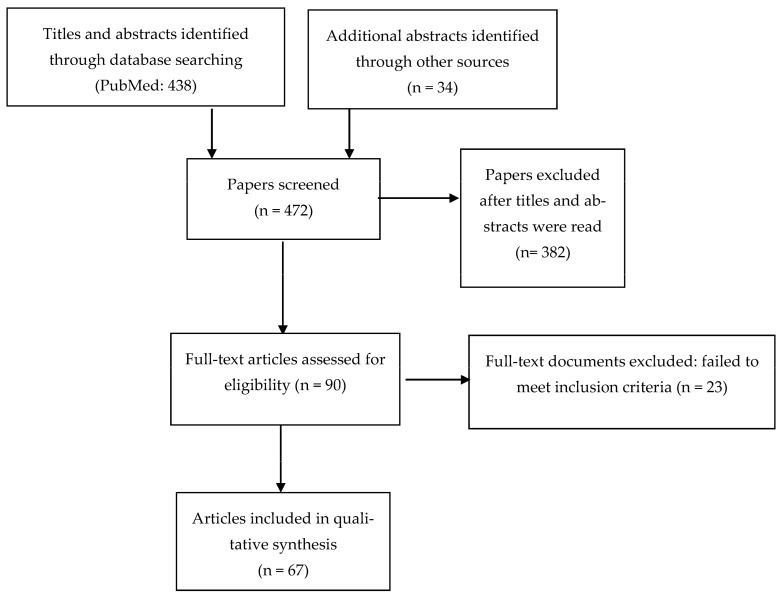
Flow diagram of included studies.

A total of 438 reports were initially screened through PubMed, and 34 additional studies were identified through other sources. From an initial total of 472 retrieved records, 67 studies were included in the narrative review. Included studies were classified according to the themes of the five observation monitoring stations. Figure 1 illustrates the screening and selection process.

Table 1 summarizes the evidence for the five main topics and the specific interventions resulting from the evidence.

### 3.1. Somatic Morbidity and Mortality

#### 3.1.1. Mortality in Women with Schizophrenia

Patients suffering from schizophrenia show an increased rate of premature death compared to the general population [13]. High mortality rates in the context of schizophrenia have been found for cardiovascular disease, respiratory illness, and cancers [13] and are attributable to variables linked to the disorder itself and to its treatment, as well as variables linked to societal stigma and to inadequate liaisons between psychiatry and the rest of medicine [14]. A recent meta-analysis found that the excess mortality in schizophrenia is associated with factors that are theoretically modifiable, suggesting that prevention and early management will reduce mortality risk [15].

A recent population-based cohort study investigated excess mortality in 46,896 people diagnosed with schizophrenia and 20,651 patients with non-affective psychoses other than schizophrenia [13]. The study was conducted in Hong Kong from January 2006 to December 2016. Mortality ratios were standardized for all causes, and the average life expectancy for each mortality cause was recorded. Both groups of patients showed higher all-cause, natural-cause, and unnatural-cause mortality rates compared to the general population. The majority of deaths in the total sample were attributed to respiratory diseases, cardiovascular diseases, and cancers. Life expectancy for men with schizophrenia was cut by 9.53 years, while a decrease of 8.07 years was observed for women. This is in line with a longitudinal linkage study from the Danish Psychiatric Research Register and the Danish Cause of Death Register conducted from January 1980 to December 2010 [16]. The results confirmed that patients with schizophrenia showed an earlier age of death compared to the population at large independent of death due to self-harm. Crump and collaborators found similar results [17]. As part of a national Swedish cohort study of 8277 patients with schizophrenia followed from 2003 to 2009, Crump et al. addressed the association between schizophrenia, mortality, and comorbidities. Men with schizophrenia died 15 years earlier than their peers and women 12 years earlier [17], ischemic heart disease and cancer being the leading causes of death. Within the group of patients who died from ischemic heart disease or cancer, patients with schizophrenia were diagnosed significantly later in the course of their disease. Schizophrenia plus death from cardiovascular disease or cancer was more strongly linked among women and among employed individuals. Not being on antipsychotic treatment was found to be associated with high mortality. A similar register-based nationwide study of patients with schizophrenia in Finland (1972–2015) [18] determined that several factors were able to reduce all-cause mortality: use of antipsychotics, use of lipid-modifying agents, antidepressants, and lithium. Cardiovascular disease and liver and kidney disease were the most common correlates of mortality; this was followed by a long duration of hospitalization, a history of substance abuse, and the use of benzodiazepines.

Other studies have confirmed that people with psychotic disorders who have been hospitalized have an increased risk of death from cardiovascular disease. Laursen et al. [19] studied schizophrenia outcomes in Denmark, Sweden, and Finland by calculating specific standardized mortality rates for each specific subgroup of mortality. They found higher than normal death rates in schizophrenia in both men and women, with life expectancy 11 to 20 years shorter than that found in the general population. Using Clinical Record Interactive Search software 2008, Chang and collaborators [20] explored data from a large electronic database of patients from south-east London. This group again investigated standardized mortality ratios and causes of death by diagnosis and gender in two periods of time: 2013–2017 and 2008–2012. They found that life expectancy in men and women was longer in 2013–2017 than in 2008–2012. 

A population register prospective cohort study on schizophrenia patients in Hungary [21] addressed mortality and its association with comorbid somatic conditions. A total of 65,169 schizophrenia patients were compared with a group of 325,435 control subjects. Men with schizophrenia had a shorter life expectancy than controls by 11.5 years, while life expectancy in women was shorter by 13.7 years. The gap was attributed to inadequate access to prompt medical care, lifestyle issues such as smoking, and stigma and social exclusion. 

A prospective longitudinal study, part of the Quebec Integrated Chronic Disease Surveillance System of the Public Health Agency of Canada, explored the mortality gap between psychiatric patients and others [22]. The results indicated reduced life expectancy in psychiatric patients. Cardiovascular disease and cancer were the most common causes of premature death in this study, and life expectancy was found to be 12 years lower in men (and 8 years lower in women) compared to the general population. In line with these results, a study from western China [23] found life expectancy in schizophrenia to be 52.8 years in men and 59 years in women; potential years of life lost due to disease was higher in men than women. Promotion of physical health maintenance was recommended.

A recent register-based study by Talaslahti and collaborators investigated mortality and causes of death in patients with schizophrenia whose onset age was 60 or more and patients with an onset below age 60 [24]. The follow-up period covered 10 years, between 1999 and 2008. Standardized mortality rates were higher in the group of patients with late onset than in those with earlier onset. Rates were higher in men than in women, and in the late-onset group, accidents, respiratory diseases, dementia, neoplasms, and circulatory diseases were the most common causes of death. The investigators’ conclusion was that effective collaboration between psychiatry and primary care was crucial if the toll of excess mortality was to be reduced. 

In summary, the literature agrees that mortality is premature in schizophrenia, although male/female comparative rates vary among studies. The incidence of some diseases, cancer being the best example, is notably not higher than normal in schizophrenia and related mental illness. Toender and colleagues [25] investigated incidence, disease stage at diagnosis, and mortality in patients with and without severe mental illness (SMI) using Danish nationwide registries between the years 1978 and 2013. Cancer incidence was actually lower in men with SMI than in those who were SMI-free; in women, there was no difference. This implies that high mortality is theoretically preventable in this population, at least for cancer.

#### 3.1.2. Cardiovascular Disease in Women with Schizophrenia

Cardiovascular disease is the major human cause of mortality and affects people with schizophrenia more than others because of stress, inflammation, poor diet, excessive smoking and alcohol use, sedentary lifestyles and obesity, and the metabolic effects of antipsychotics [26]. There may also be more fundamental reasons. Pillinger and collaborators investigated the association between polygenic risk scores for schizophrenia and cardiac phenotype in 32,279 patients who completed cardiac magnetic resonance imaging (MRI) [27]. For this observational study, the investigators used data from the UK Biobank. They found that high polygenic risk scores for schizophrenia were associated with decreased cardiac volumes and increased ejection fractions, suggesting a genetic overlap between schizophrenia and some at-risk cardiac phenotypes. It is known that persons with schizophrenia are vulnerable to Type 2 diabetes not only because antipsychotic drugs can induce diabetes [28], but also because there are genetic overlaps between diabetes and schizophrenia [29,30]. Importantly, diabetes is a risk factor for cardiac disease [31].

Although several inconsistencies have been reported with regard to gender differences in cardiovascular risk factors (CVRFs) in the first episodes of psychoses, a general agreement is that CVRFs increase at menopause in women with chronic psychoses [26]. 

Primary care services are the appropriate settings to identify early CVRFs and to propose intervention strategies. Smoking cessation, a heart-healthy diet and physical activity, and regular sleep routines need to be promoted in patients with schizophrenia, and especially in women at the time of menopause [26,32]. There are also reports that men and women with severe mental illness who are at elevated risk of ST-elevation myocardial infarction (STEMI) are less likely than others to be treated with revascularization procedures [33], presumably because they are seen as unlikely to follow through on post-intervention instructions. One adverse effect of antipsychotics that is more prevalent in women than men is torsades des pointes, a form of ventricular tachycardia associated with prolongation of the QT interval on ECG [34].

In general, women’s heart symptoms are not treated as vigorously as men’s because it is well known that men are more at risk for ischemic heart disease than women. This truism can lead to higher episodes of mortality in women [35].

#### 3.1.3. Respiratory Diseases in Women with Schizophrenia

Respiratory diseases are a major cause of mortality in patients diagnosed with schizophrenia. A recent systematic review and meta-analysis included 21 studies of 619,214 schizophrenia patients and 52,159,551 controls [36]. People suffering from schizophrenia showed higher rates of chronic obstructive pulmonary disease (COPD), asthma, and pneumonia compared with the general population. The following schizophrenia prevalence rates were reported: 7.7% for COPD, 7.5% for asthma, 10.3% for pneumonia, and 0.3% for tuberculosis. The authors suggested the need to improve the prevention and management of respiratory diseases in this population in order to reduce both morbidity and mortality. No specific mention of gender differences was made. This is absolutely in line with other studies exploring respiratory health in people with psychosis. Partti and colleagues carried out a study based on a nationally representative sample of 8028 adults with psychosis, assessing lung function using spirometry [37]. Clinical data on respiratory diseases and serum cotinine level (as a measure of smoking) were quantified. Lung function outcomes were found to be lower in schizophrenia patients than they were in the general population. Schizophrenia was associated with an increased risk of pneumonia, chronic obstructive pulmonary disease, and chronic bronchitis, as well as high levels of cotinine.

More recently, Jaén-Moreno and collaborators explored the association between schizophrenia (and bipolar disorder) and chronic obstructive pulmonary disease (COPD) [38]. The prevalence of COPD was found to be between 2.6 and 52.7% in patients with schizophrenia. Advanced age was associated with COPD, and mortality from COPD was higher in schizophrenia than in the psychosis-free population. A Danish nationwide population-based cohort study from Danish registries (2008–2013) [39] explored the association between schizophrenia and quality of care in COPD patients. Patients with schizophrenia received less frequent treatment with long-acting muscarinic antagonists (LAMAs) or long-acting beta-2 agonists (LABAs) compared to patients without schizophrenia. Among COPD schizophrenia patients, women received LAMAs/LABAs more frequently than men. 

Obstructive sleep apnea (OSA) is not rare in patients suffering from schizophrenia because several risk factors are common to both clinical conditions. A recent systematic review investigated the prevalence of OSA in schizophrenia, the physical and psychiatric comorbid conditions in patients with OSA and schizophrenia, the association of antipsychotics and OSA, and the validity of screening tools [40]. The prevalence of OSA in schizophrenia was between 1.6 and 52% and was associated with male sex, age >50 years, and a body mass index of over 25. Few data on physical and mental health outcomes following the treatment of OSA are available. Focusing on women with schizophrenia, Seeman [41] found that the rate of OSA in obese women and postmenopausal women is similar to that of men. The use of antipsychotic medication, alcohol, and cigarettes plus a family history of OSA increase the risk. Adherence to the use of Continuous Positive Airway Pressure (CPAP) is difficult in this population but higher in women compared to men. 

In general, men smoke more than women and are more exposed to toxic industrial fumes, although women are more likely to develop allergic reactions to air particles [42]. Sedative antipsychotics may undermine deep breathing in both sexes [43].

#### 3.1.4. Cancer in Women with Schizophrenia

Cancer is one of the major causes of death in patients with schizophrenia. Previous research has shown that some cancers, for instance breast cancer, are more prevalent in patients with schizophrenia than in others, and other types of cancer (prostate cancers) are less prevalent [44]. A recent narrative review investigated the incidence and mortality of cancer in schizophrenia [44]. This study concluded that medical disparities exist in the treatment of patients with serious mental illnesses. Risk factors can be attributed to the disorder itself, to the treatment received, and to lifestyle factors. Low parity, low rates of lactation, obesity and sedentary behavior, caffeine, smoking, and alcohol use have been associated with a higher cancer risk. Other treatment-related factors, such as antipsychotic-induced hyperprolactinemia, are also suspected risks of breast cancer in women with schizophrenia. It is important to keep in mind that high prolactin levels can result from chronic stress [45]. Low rates of cancer screening are characteristic of schizophrenia in women and attributable to social isolation, small social networks, and the psychopathological characteristics of the illness (negative symptoms, lack of trust in recommendations, cognitive defects), as well as failure on the part of medical staff to encourage and ensure attendance at screening.

A systematic review and meta-analysis explored the mortality rates of common site-specific cancers in patients suffering from schizophrenia [46]. A total of seven studies including a total sample of 1,162,971 participants were included in the meta-analysis. Significant increases in the mortality risk of breast cancer and colon cancer were found in schizophrenia compared to the general population, independent of sex. For women, lung and colon cancer mortality were significantly increased. 

An observational cohort study investigated psychiatric morbidity (including schizophrenia) and non-participation in breast cancer screening [47]. The cohort included 144,264 women, and it was found that psychiatric morbidity correlated with non-participation. Along the same lines, Woodhead and collaborators carried out a population-based study from the London borough of Lambeth (UK), linking primary and secondary care data with the goal of comparing breast and cervical screening in women suffering from severe mental illness [48] with that of primary care patients without psychiatric illness. A diagnosis of schizophrenia and symptom severity were associated with low rates of breast and cervical cancer screening, suggesting that physicians do not recommend extra procedures with severely ill patients, either because they do not want to increase their distress or because they think appointments will not be kept and that the resulting instructions will not be followed.

### 3.2. Hyperprolactinemia

Although prolactin is mainly a hormone involved in processes of reproduction and lactation, it is also synthesized in response to stress [49]. Hyperprolactinemia is a well-known adverse effect of antipsychotic medications (some more than others); however, it may also result from the stress of acute schizophrenia symptoms [49,50,51]. 

A recent study by Ittig and collaborators, a part of the prospective Früherkennung von Psychoses (FePsy) study [50], investigated prolactin levels in drug-naïve patients who were not taking any medication that influenced the secretion of prolactin. A total of 116 antipsychotic naïve at-risk mental state (ARMS) individuals and 49 first episode of psychosis (FEP) patients were recruited. After correction for potential confounding factors, 32% of ARMS patients and 35% of FEP patients showed evidence of hyperprolactinemia. Statistically significant differences in prolactin levels between ARMS and FEP individuals were not found. These findings have been replicated in subsequent studies. Delgado-Alvarado and collaborators, as a part of the FAPIP study, explored prolactin levels in 270 antipsychotics-naïve FEP patients and 153 healthy controls [51]. Prolactin levels were higher in women than men, and these levels were also higher in antipsychotic-naïve FEP patients compared to healthy controls, which suggests stress induction. In the context of the European First Episode Schizophrenia Trial (EUFEST), Riecher-Rössler et al. confirmed that hyperprolactinemia is present in patients with schizophrenia in the early stages of disease and can mediate, by way of estrogen reduction, the pathway from stress to the induction of psychotic symptoms [51].

Hyperprolactinemia may have important short-term and long-term clinical consequences. A review of the effects of antipsychotics on prolactin levels and menstruation in women concluded that regular menses play an important role in reproductive and physical health, while hyperprolactinemia interferes with both menstrual regularity and fertility [52]. This research team recommends monitoring menses and prolactin levels and preventing hyperprolactinemia in order to avoid short- or long-term consequences. One of these consequences may well be breast cancer. Taipale and collaborators, as a part of the Finnish nationwide register of patients in hospital treatment, carried out a case–control study investigating the effect of prolactin-raising antipsychotics on the risk of breast cancer in schizophrenia populations [53]. Cases included women with schizophrenia and breast cancer who were matched with schizophrenia women free of breast cancer. A total of 30,785 women were recruited between 1972 and 2014, and, by 2000–2017, 1069 had been diagnosed with breast cancer. The finding was that 1 to 4–5 years of cumulative exposure to prolactin-sparing antipsychotics (clozapine, quetiapine, or aripiprazole), i.e., small amounts of extra prolactin, did not increase the risk for breast cancer. On the other hand, exposure to 5 or more years of prolactin-raising antipsychotics was significantly associated with an increased risk. Chu and collaborators also investigated the risk of breast cancer associated with antipsychotics using a public healthcare database in Hong Kong [54]. They found a significant association between the use of first-generation antipsychotics and breast cancer in women with schizophrenia and bipolar disorder. Second-generation antipsychotics, on the other hand, were associated with breast cancer only in women with bipolar disorder, not schizophrenia. The conclusion was that further research is needed to clarify the discrepancy. A recent review on the topic has highlighted extensive methodological difficulties in this field of research [55]. Some of the variables include the many risk factors for breast cancer to which these women are exposed and the uncertainty about which antipsychotics elevate prolactin, and to what degree. The investigators advocate transparency about what is known and unknown with regard to risks and recommend obtaining written consent when antipsychotics are prescribed.

Osteoporosis and low bone mineral density (BMD) are frequently seen in patients with schizophrenia. Kinon and colleagues [56] carried out a cross-sectional study to determine the prevalence of low bone mass density (BMD) in 402 patients with elevated prolactin who had been prescribed risperidone or first-generation antipsychotics. After three months, BMD was assessed using ultrasonography of the calcaneus. They found that 25% of women and 33% of men had low BMD. The investigators found that hyperprolactinemia induced by prolactin-raising antipsychotics was associated with low bone mass more in men than in women. These findings are in agreement with a subsequent study by Lin and collaborators [57]. A total of 80 men and 115 women suffering from schizophrenia were included as study participants. Blood levels of prolactin and sex and thyroid hormones were all associated with bone mineral density as measured by a dual-energy X-ray absorptiometer. Again, this was more pronounced in men. The issue here is menopause. Menopause and the associated decline in estrogen heightens the risk of osteoporosis for women, especially low-weight women because estrogen is synthesized in adipose tissue. The effect of antipsychotics on BMD in men and women depends essentially on estrogen levels. High prolactin levels can be elevated by a number of factors, among them the type of antipsychotic and its dose and duration. Increased prolactin decreases estrogen production, which, in turn, lowers bone density. Prior to menopause, this affects men more than women. After menopause, more women are affected [58].

More recent studies have investigated the use of prolactin-raising antipsychotics in terms of the rate of low-energy fractures in people with schizophrenia. Solmi and collaborators conducted a nested case–control study using a Finnish nationwide register [59]. Individuals suffering from schizophrenia aged 18–65 years with low-energy fractures (LEFs) were compared with matched controls with schizophrenia but without LEFs. Antipsychotics were divided into two groups: prolactin-sparing antipsychotics and prolactin-raising antipsychotics. Long-term exposure to prolactin-increasing antipsychotics *at any dose*, and also high cumulative doses of prolactin-*sparing* antipsychotics were associated with significantly increased odds of LEF. This study concludes that all antipsychotics, to varying degrees, raise prolactin levels [59].

Because prolactin lowers both estrogen and testosterone production, hyperprolactinemia is associated with infertility in men as well as in women. Edinoff and collaborators [60] recommend asking women about their wishes with respect to pregnancy prior to prescribing psychotropic medications. Sexual function is also affected by prolactin. Rubio-Abadal and collaborators [61] conducted a cross-sectional study of persons with schizophrenia and related disorders who attended mental health services. The Positive and Negative Syndrome Scale (PANSS) was used to assess psychotic symptoms, the Personal and Social Performance Scale (PSP) was used for general functioning, and the Changes in Sexual Functioning questionnaire (short form) was used to evaluate sexual dysfunction. From a total sample of 101 patients, 30 women were recruited for the study. Hyperprolactinemia was present in 71.3% of the sample, and sexual dysfunction was significantly higher in these women than in those with prolactin levels within the normal range. No sex differences were found in the prevalence of hyperprolactinemia or of sexual dysfunction. 

In summary, antipsychotic medication used to treat schizophrenia elevates prolactin levels, with some drugs doing so more than others. High prolactin levels over long stretches of time result in a number of adverse health effects, some affecting women more than men at different times of the life cycle.

### 3.3. Substance Use Disorders

Comorbidity between psychiatric disorders and substance use disorders occurs frequently [62]. Gurriarán and collaborators carried out a genome-wide association study of patients with schizophrenia and other severe mental illnesses with the aim of finding correlations between polygenic scores (PGSs) and addictions [62]. PGSs were ascertained for 534 patients fulfilling DSM-IV criteria for the following substance use disorders: alcohol, tobacco, cannabis, hypnotics, stimulants, hallucinogens, cocaine, and opiates. Schizophrenia polygenic scores were higher in women with alcohol abuse than in their male peers. Overall, similar PGS values for schizophrenia led to less of an association with substance abuse disorders in women than in men. In other words, despite identical underlying genetic risk for schizophrenia, men and women are not similarly predisposed to develop a dependency on addictive substances. Men’s susceptibility must depend on something more than PGSs.

Women differ in many ways from men with respect to substance use disorders. Casanovas and colleagues [63] found that all substance abuse disorders were less prevalent in women than in men, though it is possible that they may be underdiagnosed in women. Their use, especially in the case of cannabis, though lower than in men, impacts women’s health more negatively. Ayesa-Arriola and collaborators [64] explored clinical, cognitive, premorbid, and sociodemographic characteristics in a sample of 209 first-episode psychosis patients who were assessed at baseline and again 1, 3, and 10 years later. At follow-up, men were more likely than women to be diagnosed with schizophrenia plus either cannabis or alcohol use disorders. 

It has been hypothesized that the association between schizophrenia and co-occurring substance use disorders is mediated by dysfunction in brain reward circuitry [65]. A recent review on the topic highlighted the fact that patients receiving clozapine had lower rates of substance use than those treated with other second-generation antipsychotics [65].

The use of hypnotic medication in patients suffering from schizophrenia has been a focus of interest in recent years. A nationwide cross-sectional study, part of the Effectiveness of Guidelines for Dissemination and Education in Psychiatric Treatment (EGUIDE) project, analyzed results from 2146 inpatients with schizophrenia [66]. The use of all psychotropic drugs was recorded, and it was found that 55.7% of schizophrenia patients were prescribed at least two hypnotics, with 17.6% taking more than two hypnotic agents. No sex differences were reported.

Steingrímsson and collaborators compared differences in mortality rates (1983–2007) in Icelandic psychiatric patients according to hospital discharge diagnosis [67]. A total of 14,281 patients were included. The findings were that mortality rates for men and women with substance use disorders were similar to rates in schizophrenia patients with no history of substance use disorders. In other words, premature death was equally common for those two diagnoses, suggesting that comorbidity between the two further reduces the already short lifespan of schizophrenia.

Co-existing substance use disorders have long been associated with a greater severity of psychopathological symptoms in women with psychosis. Aakre and colleagues compared rates of trauma and posttraumatic stress disorders in three groups of patients at risk for psychological trauma: women with schizophrenia and comorbid substance use disorders (*n* = 42), women with non-psychotic depression and substance use disorders (*n* = 38), and women with substance use disorders without any other major psychiatric disorder (*n* = 37) [68]. Posttraumatic stress disorder was evaluated by using the clinician-administered version of the Clinician-Administered PTSD Scale. Symptoms were assessed by the Positive and Negative Syndrome Scale (PANSS), and the Traumatic Life Events Questionnaire (TLEQ) was used to evaluate trauma in both childhood and adulthood. Women with schizophrenia and comorbid substance use disorders experienced more trauma than women with substance use disorders alone. 

The comorbidity of substance use disorders and schizophrenia is particularly important for women in the perinatal period. Fabre and collaborators [69] conducted a population-based cohort study between 2015 and 2019 focusing on the effects of substance use on labor and delivery. The sample consisted of women with schizophrenia and control women. As a group, the women with schizophrenia were older and consumed more cigarettes, alcohol, and substances than controls. Schizophrenic women also suffered more frequently from obesity, diabetes, and chronic obstructive pulmonary disease. They also experienced significantly more pregnancy and delivery complications. 

Since substance abuse affects not only the health of women but also that of their children, it becomes an especially important treatment target when treating women with schizophrenia.

### 3.4. Social Exclusion and Discrimination

Jeste and collaborators [70] reported recently on the impact of early-life adversities, poverty, social exclusion, discrimination, racism, food insecurity, and residence in disadvantaged neighborhoods on the course of schizophrenia. Social factors significantly impact physical and mental health and longevity in schizophrenia, although the pathophysiology of their influence is unclear. Hormones and immune/inflammatory processes are reportedly involved, and these differ markedly between men and women, which led our team to specifically review the impact of social risk factors on women [71]. We found that, in the domains of childhood and adult abuse or trauma, victimization, stigma, housing, and socioeconomics, women with schizophrenia suffered substantially more negative consequences than male peers.

A recent study by Das-Munshi and collaborators investigated the effects of ethnicity and deprivation on life expectancy in people suffering from schizophrenia [72]. Irrespective of ethnicity, this research team found reductions in life expectancy in people with schizophrenia even when compared to residents of the most deprived areas in England who were schizophrenia-free. Social exclusion and marginalization were held responsible [72].

Carliner and colleagues reviewed the association between cardiovascular risk factors in racial and ethnic minorities living with schizophrenia [73] and found that women in particular suffered from an increased risk of cardiovascular risk factors compared to the general population. 

Farrelly et al. carried out a cross-sectional study investigating anticipated and experienced discrimination in 202 individuals from south London (47% with schizophrenia) [74]. Approximately 93% of the sample confirmed *anticipating* discrimination and 87% had actually experienced discrimination in the year prior to the study. Individuals of mixed ethnicity reported the highest levels of experienced discrimination. Women were more likely than men to anticipate discrimination, and this was linked with worsening clinical symptoms such as suspiciousness, anxiety, and depression.

Existing research on social factors that affect women is highly variable methodologically, so conclusions must remain tentative. Study design is mostly cross-sectional; longitudinal studies in this area are scarce and sample sizes are relatively small.

### 3.5. Drug Safety

Many pharmacological studies have shown that men and women metabolize drugs differently. There are sex differences in drug absorption, distribution, liver and gut metabolism, elimination, protein binding, drug interactions, brain blood flow, and action at target sites [75,76,77]. There are also sex/gender differences in drug regimen adherence and in the tolerance of side effects. For some drugs, these differences mean that doses need to differ in order for the drug to be both effective and safe. Dosing guidelines do not usually take sex/gender differences into account, but this issue can become clinically important when prescribing antipsychotic medication, especially during times of rapid hormonal or lipid store change, e.g., postpartum, at menopause, or during sudden weight loss [76]. As mentioned earlier, drug-induced prolactin highs and weight gain can also lead to different consequences in men and women [78].

Clozapine is an atypical antipsychotic medication used for otherwise treatment-resistant schizophrenia patients, a group that represents about 20 to 30% of patients with schizophrenia [79]. As a part of the South London and Maudsley retrospective cohort study, Wellesley Wesley and collaborators investigated the effect of gender on clozapine prescription for treatment-resistant schizophrenia [80]. Clozapine was prescribed for this indication in 77% of women compared to 85% of men. There may be good reasons for this discrepancy, such as women refusing clozapine because of its risk during a potential pregnancy, because of the weight gain risk, or because its use necessitates frequent hospital or clinic visits for bloodwork. However, it can also mean that women are receiving inadequate care for their condition.

Adherence to psychotropic medications is crucial to maintain clinical stability and to avoid problems related to the severity and chronicity of symptoms. In the context of women with schizophrenia, as earlier published by our group, therapeutic drug monitoring and adherence programs are recommended. Long-acting injectable antipsychotic medications can be offered in certain cases with the aim of ensuring adherence and avoiding overdoses [1]. 

Women with schizophrenia who are on contraceptive medication, those who show premenstrual symptom exacerbation or are pregnant, those who are postpartum or lactating, and those undergoing menopause all require antipsychotic drug adjustments due to changed hormonal milieu [81,82]. At the time of menopause, the use of long-acting injectable antipsychotics eludes first-pass liver metabolism and, thus, overcomes the effect of estrogen loss on the action of estrogen-dependent liver enzymes, subsequently preventing raised doses and concomitant adverse effects [11]. Consultation and staff re-training may be necessary to provide up-to-date pharmacological expertise.

## 4. Discussion

As a result of this literature review, several specialized interventions are proposed for women with schizophrenia.

The elevated risk of premature death attributed to cardiovascular disease, respiratory disease, and cancers [13,15] calls for targeted interventions to improve the physical health of all patients with schizophrenia, including women. Clinical decision tools have been shown to be useful in this regard, realizing a 4% risk reduction versus controls [83]. Chronic obstructive pulmonary disease is associated with patient mortality in this population [38] and requires specific programs for screening and early intervention, as does obstructive sleep apnea [40]. The implementation of outpatient cardiorespiratory fitness groups and close collaboration between psychiatrists and primary care physicians are indicated [84,85,86,87]. 

A recent systematic review and meta-analysis reported the prevalence of chronic obstructive pulmonary disease (COPD) and chronic bronchitis in non-psychotic populations [88]. Forty-three studies were included, and the estimated prevalence of COPD was found to be 11.1% (CI: 7.4–14.8), which is similar to that of schizophrenia, suggesting that smoking and breathing bad air are universal. Population studies report that childhood asthma is more common in boys, while adult asthma is more common in adult women [89]. That may be due to the propensity of women developing allergies to medications and may account for the problems found in women with schizophrenia.

Vancampfort and collaborators designed a 10 h workshop to improve knowledge and confidence in physical activity prescription in health professionals [90]. Outpatient groups intended for women may require special consideration, such as consideration of transportation costs and childcare issues. 

A recent systematic review and meta-analysis found that patients with schizophrenia show a significantly high risk of mortality from breast, colon, and lung cancer [46]. Instituting preventive strategies when possible and educating patients, as well as sometimes accompanying patients to cancer screening appointments, ensures early detection. Volunteers, family members, and peer support workers can be called in to help with these important tasks [91]. Specific collaboration programs with other medical specialties, such as gynecology, gastroenterology, respiratory medicine, and oncology, will ensure compliance. The big killer in women is breast cancer; early detection is vital [48], but so is close monitoring and follow-ups of adherence to treatment protocols.

One way of protecting against breast cancer is to prescribe prolactin-sparing antipsychotics to women with schizophrenia while keeping doses as low as possible [53]. Other ways include encouraging healthy diets and physical activity to prevent obesity. Reducing prolactin also helps with sexual dysfunction and fertility in premenopausal women and helps to prevent osteoporosis after menopause. A recent systematic review and meta-analysis examined evidence from open-label studies and randomized clinical trials on four prolactin-lowering strategies in patients suffering from psychotic disorders [92,93]. The following strategies were considered: (1) adding aripiprazole to the antipsychotic regimen, (2) switching altogether to prolactin-sparing antipsychotics, (3) adding dopamine agonists (e.g., cabergoline), and (4) adding metformin. The authors concluded that adding aripiprazole was the best first option.

Whereas substance abuse is more of a problem for men with schizophrenia than for women, consequences for women can include victimization and homelessness [68]. Schizophrenic women with substance use disorders are at a high risk of traumatic life events and often present with comorbid posttraumatic stress disorder. Early treatment of trauma is often required [67,68].

Health promotion interventions have been found to be effective in reducing the risk of mortality. Westman and collaborators carried out an intervention trial testing the efficacy of a health promotion intervention targeting 570 patients with psychosis [94]. Body mass index, waist circumference, heart rate, and general health improved during the run-in period, suggesting a positive effect on cardiometabolic risk factors. Health promotion is also important in combating excessive substance use. 

Reduced use of hypnotic medications is an important goal because (a) these drugs are potentially addictive and (b) they may contribute to the negative symptoms of schizophrenia. They may also contribute to respiratory problems [69].

We found that expected and perceived discrimination in people with schizophrenia differed between men and women [74]. Women were more likely to anticipate discrimination than men and, therefore, to internalize it. Group discussions about stigma and how best to deal with it are helpful to patients with schizophrenia. The kinds of stigma associated with women (“women like these should not be allowed to be mothers”) need to be included [95,96,97]. 

Homelessness is a major social problem that impacts mental health and well-being; it is caused by several significant contributory factors [98]. A recent umbrella review found high prevalence rates of depressive and anxiety disorders, schizophrenia spectrum, and other psychotic disorders in homeless people [98]. The prevalence of schizophrenia is very frequent in homeless populations [99]. Tinland and collaborators investigated the proportion of schizophrenia and bipolar disorder [100] in homeless populations and found that women suffered disproportionally because of physical and sexual assault, subsequent PTSD, and depression, leading to increased suicide risk and deteriorations in physical health. This appears to justify the specific observatory for social exclusion and discrimination in our mental health unit for women.

Barriers to appropriate women-specific and reproductive stage-specific choices and doses of antipsychotics include inadequate knowledge and skills. Certain antipsychotic medications (such as clozapine) and long-acting antipsychotic injections are underused in women [101]. Training on how to initiate new regimens when indicated and recognize and deal with potential side effects is a critical need [96]. Regular checks for side effects of most concern to women (thyroid tests, waist circumference measures, mammography, cervical screening, diabetes screen, EKG) need to be mandatory in psychiatric services for psychosis [102]. 

Our narrative review has several limitations, as well as strengths, that need clarification. It is not a systematic review; pertinent publications not referenced in the major medical database used (PubMed) may unintentionally have been overlooked. Papers published in the many languages unfamiliar to any of the authors were not included, so the literature search cannot be considered global. A major strength is that, to the best of our knowledge, this is the first review that brings together most of the knowledge pertaining to five important clinical issues faced by women with schizophrenia, and, in response to that knowledge, the study suggests specific interventions capable of improving the health outcomes of this population. 

## 5. Conclusions

We have reviewed issues of concern to the health and welfare of women with schizophrenia. We describe how, in our own service, we have instituted practices that help to monitor five such areas of concern. We focus on somatic issues (respiratory and cardio-metabolic comorbidities, cancer, organic consequences of hyperprolactinemia and excessive substance use), social issues such as marginalization, exclusion, and negative bias, and, finally, differences in the pharmacokinetics and pharmacodynamics of antipsychotic drugs in women versus men. We point out that women’s medications (in terms of dose and perhaps mode of administration) need to change over the course of successive life stages. In conclusion, we emphasize that there are effective ways of training staff to monitor these important clinical issues, as well as ways of instituting preventive and early interventive measures. 

## Figures and Tables

**Table 1:** Evidence of women’s specific needs.

	Findings	Specific Interventions
**Somatic morbi-mortality**	Schizophrenia mortality rates up due to cardiovascular, respiratory, or oncological illness	Physical exercise/cardiorespiratory groups Liaison with primary care for metabolic syndrome, cancer screening, and the use of metformin
	Cardiovascular risk associated with lifestyle	Psychoeducation patients and family
	Risk of pneumonia and chronic obstructive pulmonary disease	Close liaison with primary care
**Hyperprolactinemia**	Hyperprolactinemia associated with amenorrhea, galactorrhea, and fertility loss	Strategies to lower prolactin levels
	Hyperprolactinemia contributes to sexual dysfunction, increased breast cancer risk, and osteoporosis	Appropriate screening and staff training
**Substance use disorder**	Men exhibit more substance use disorders but consequences are different in women	Trauma-focused prevention
**Discrimination, social exclusion**	Social and medical stigma related to sex and reproduction in women	Staff discussion and training
**Prescription and drug safety**	Clozapine use in women Drug choice and dosing for women over the life cycle	Pharmacovigilance, training

## Data Availability

The data presented in this review are available upon request from the corresponding author.
